# The impact of inorganic fillers, organic content, and polymerization mode on the degree of conversion of monomers in resin-matrix cements for restorative dentistry: a scoping review

**DOI:** 10.1007/s00784-024-05829-6

**Published:** 2024-07-27

**Authors:** Marcionilia Santos, Rita Fidalgo-Pereira, Orlanda Torres, Oscar Carvalho, Bruno Henriques, Mutlu Özcan, Júlio C. M. Souza

**Affiliations:** 1 Oral Pathology and Rehabilitation Research Unit (UNIPRO), University Institute of Health Sciences (IUCS), CESPU, 4585-116 Gandra PRD, Portugal; 2 Center for Interdisciplinary Research in Health (CIIS), Faculty of Dental Medicine (FMD), Universidade Católica Portuguesa (UCP), 3504-505 Viseu, Portugal; 3 Center for MicroElectroMechanical Systems (CMEMS-UMINHO), University of Minho, Campus Azurém, 4800-058 Guimarães, Portugal; 4 Associate Laboratory (LABBELS), University of Minho, 4710-057 Guimarães, Braga Portugal; 5 Ceramic and Composite Materials Research Group (CERMAT), Federal University of Santa Catarina (UFSC), Campus Trindade, Florianópolis, SC 88040-900 Brazil; 6 Clinic for Masticatory Disorders and Dental Biomaterials, Center of Dental Medicine, University of Zurich, 8032 Zurich, Switzerland

**Keywords:** Restorative dentistry, Resin cements, Polymerization, Degree of conversion

## Abstract

**Purpose:**

The main aim of the present study was to carry out a scoping review on the differences in degree of conversion of monomers regarding several types resin cements, indirect restorative materials, and light-curing procedures used in dentistry.

**Method:**

A bibliographic review was performed on PubMed using the following search items: “degree of conversion” OR “filler” AND “resin cement” OR “inorganic cement” AND “organic” OR “radiopacity” OR “refractive” OR “transmittance” OR “type” AND “resin composite.” The search involved articles published in English language within the last thirteen years. A research question has been formulated following the PICO approach as follow: “How different is the degree of conversion of monomers comparing several types of resin-matrix cements?”.

**Results:**

Within the 15 selected studies, 8 studies reported a high degree of conversion (DC) of the organic matrix ranging from 70 up to 90% while 7 studies showed lower DC values. Dual-cured resin-matrix cements revealed the highest mean values of DC, flexural strength, and hardness when compared with light- and self-polymerized ones. DC mean values of resin-matrix cements light-cured through a ceramic veneer with 0.4 mm thickness were higher (~ 83%) than those recorded for resin-matrix cements light-cured through a thicker ceramic layer of 1.5 mm (~ 77%).

**Conclusions:**

The highest percentage of degree of conversion of monomers was reported for dual-cured resin-matrix cements and therefore both chemical and light-induced pathways promoted an enhanced polymerization of the material. Similar degree of conversion of the same resin-matrix cement were recorded when the prosthetic structure showed a low thickness. On thick prosthetic structures, translucent materials are required to allow the light transmission achieving the resin-matrix cement.

**Clinical relevance:**

The chemical composition of resin-matrix cements and the light-curing mode can affect the polymerization of the organic matrix. Thus, physical properties of the materials can vary leading to early clinical failures at restorative interfaces. Thus, the analysis of the polymerization pathways of resin-matrix cements is significantly beneficial for the clinical performance of the restorative interfaces.

## Introduction

In contemporary dental practice, resin-matrix cements are routinely used for bonding prosthetic structures such as prosthetic crowns, multi-unit prostheses, veneers, onlays, inlays, and fiber-reinforced posts [1–4]. However, clinical failures on resin-matrix cements have been reported considering the change of color, marginal leakage, microstructure, and fracture at interfaces [3, 5]. The cementation of indirect restorations over tooth tissues also depends on the surface modification of the inner surface of the indirect restorations by grit-blasting, chemical conditioning, and acid etching [6, 7]. Clinical issues have been associated with several factors including mainly the type of resin-matrix cement, type of prosthetic structures, prosthetic restoration to surrounding tissues, light-curing unit, and polymerization mode. Thus, an insufficient polymerization of the resin-matrix cement has become a major concern taking into account the required physical properties for long-term performance in the oral cavity [6–9].

Resin-matrix cements are mainly composed of an organic matrix embedding silanized inorganic fillers [2, 5]. The organic matrix of resin-matrix cements comprises a mixture of monomers such as bisphenol-A glycol dimethacrylate (Bis-GMA), urethane dimethacrylate (UDMA), and triethylene glycol dimethacrylate (TEGDMA) [5, 10–13]. Chemical agents (i.e., peroxides) and photoinitiators systems (i.e., camphorquinone combined with tertiary amine) are key compounds to initiating the cross-linking among chain of monomers leading to the polymerization [14]. Depending on the photoinitiatior, the cross-linking of monomers is stimulated by light irradiance with a wavelength ranging from 360 up to 500 nm [4]. However, monomers with a high molecular mass usually exhibit low mobility that contributes to a lower shrinkage although it increases the viscosity of the cement and decreases the degree of conversion (DC) of monomers [4, 15]. Low viscosity monomers such as TEGDMA have the purpose of decreasing the viscosity allowing the flowability of the resin-matrix cement [16]. The optical and mechanical properties of the resin-matrix cements are tailored from the reinforcement of the organic matrix with micro- and nano-scale inorganic fillers such as zirconia, zirconium silicate, barium glass, ytterbium fluoride, and/or amorphous silica [13, 17].

The percentage of DC usually represents the amount of polymerized double carbon bonds that were converted into single bonds in the carbon chain [10, 18]. The DC percentage of resin-matrix cements generally ranges from 52 up to 75% [19–21]. Both extrinsic and intrinsic material factors influence DC, namely the monomers nature, inorganic particles, and photoinitiators [10]. Thus, other extrinsic factors involving polymerization pathways can affect DC. In order to have a correct DC the distance between the light curing (LCU) unit and indirect restoration must be below 8 mm, the LCU must guarantee the correct irradiance and the time exposure must be sufficient for the polymerization reaction to correctly occur. Other parameters must also be ensured, namely, the resin cement thickness, the indirect restoration thickness, and the compatibility between the wavelength of LCU and photoinitiator system [22, 23].

Previous studies have reported a correlation between DC percentage and physical properties of the resin-matrix cement. Indeed, a high DC percentage is correlated with enhanced physical properties as reported by high mean values of flexural strength, elastic modulus, fracture toughness, and hardness [19–21]. Indeed, the hardness measurement is often used as an indirect method to measure the polymerization efficiency [24]. The increase of the monomers’ cross-linking over light-curing can be monitored by the elastic modulus and hardness acquired by micro- and nano-indentation assays [10, 25].

A low DC percentage is a result from failure on the polymerization of the resin-matrix cement leading to poor chemical and physical stability [20]. For instance, insufficient polymerization of the resin-matrix cement can promote the chemical reactivity of the material with the surrounding environment leading to changes in optical properties [11]. A low degree of conversion can promote detrimental effects to the physical properties of the materials, namely water sorption solubility, low strength and hardness [23]. Another issue that could lead to toxicity is the excessive amount of resin cement that can be trapped into surrounding tissues. Thick layers of resin cement debris that are not correctly polymerized can cause inflammatory reactions to the surrounding tissues including the dentin-pulp complex [26]. Additionally, the mechanical properties of the material are negatively affected and therefore the resin-matrix cement becomes susceptible to cracks and catastrophic fracture at the restorative interface.

The main aim of the present study was to carry out a scoping review on the differences in degree of conversion of monomers regarding several types of resin cements, indirect restorative materials, and light-curing procedures used in dentistry. It was hypothesized that the degree of conversion of monomers varies depending on the inorganic fillers, organic matrix of the resin cements, types of indirect restorative materials and polymerization pathways.

## Method

### Search strategy

A bibliographic review was performed on PubMed (via National Library of Medicine) considering that includes the major journals in the field of dentistry and biomaterials. The present search of studies was carried out in accordance with previous integrative review articles [23, 26–29]. The following search terms were assessed: “degree of conversion” OR “filler” AND “resin cement” OR “inorganic cement” AND “organic” OR “radiopacity” OR “refractive” OR “transmittance” OR “type” AND “resin composite.” Combination of terms were assessed regarding the purpose of this study. Also, a hand-search was performed on the reference lists of all primary sources and eligible studies of this integrative review for additional relevant publications. The inclusion criteria encompassed articles published in the English language from January 2011 up to January 2024, focusing on the degree of conversion of the organic matrix of resin-matrix cements. The eligibility inclusion criteria used for article searches also involved in vitro studies; randomized controlled trials; animal assays; and prospective cohort studies. The exclusion criteria were the following: papers without abstract; case report with short follow-up period; reviews; pilot studies; studies on the effect of fillers through other composite materials applied in different biomedical or engineering fields. Studies based on publication date were not restricted during the search process. A research question has been formulated following the PICO (Population, Intervention, Comparison, and Outcome) approach as follow: “How different is the degree of conversion of monomers comparing several types of resin-matrix cements?” The following factors were taken into consideration: (i) Population: resin cements, human participants, animals, indirect restorative materials, polymerization mode; (ii) Intervention: mechanical assays, optical analyses, microscopy, chemical analyses, cementation procedures, light-curing mode, and equipment. (iii) Comparison: different resin cements, polymerization parameters, types of restorative materials. (iv) Outcomes: major findings related to the degree of conversion of different resin cements affected or not by the indirect restorative material.

### Study selection and data collection process

The selection of studies was carried out in three steps. At first, studies were scanned for relevance by title, and abstracts of the non-excluded studies were evaluated. Two researchers (MS and JCMS) independently analyzed the titles and abstracts of potentially relevant retrieved articles that met the inclusion criteria. The total of articles was compiled for each key term combination and therefore duplicates were removed using the Mendeley Reference Manager (ed. Elsevier). The second step comprised the evaluation of the abstracts and non-excluded articles, following the eligibility criteria on the abstract review. Selected articles were individually read and analyzed concerning the purpose of this study. The study selection at that step also encompassed the exclusion criteria. At last, the eligible articles received a study nomenclature label, combining first author names and year of publication. The following variables were collected for this review: authors’ names, study design/purpose, resin-matrix cements (chemical composition), restoration type, light-curing procedure, degree of conversion (%), and main outcomes. Data of the studies were harvested directly into a specific data-collection form to preventing multiple data recording within the same study (e.g., reports with different set-ups). Such evaluation was individually carried out by two researchers, followed by a joint discussion to select the most relevant studies.

## Results

**Fig. 1:**
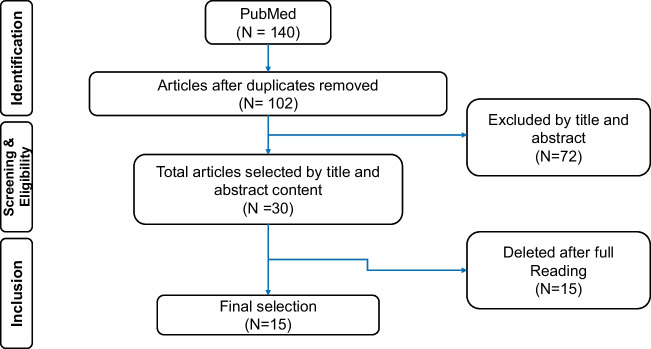
Flowchart of the search strategy used in this study

The initial search on PubMed database identified a total of 140 studies of which 38 duplicates were removed on the gradual combination of some terms (Fig. 1). The titles and abstracts of the 102 articles were read seeking concordance with the inclusion criteria of the present study. A total of 72 studies were excluded concerning they did not meet the inclusion criteria. The evaluation of titles and abstracts resulted in 30 potentially relevant studies, although 15 studies were excluded because they did not provide comprehensive data taking into account the purpose of this review study. Thus, 15 studies were included in this review (Fig. 1).

Within the 15 selected studies, 8 studies (53%) reported a high degree of conversion (DC) of the organic matrix ranging from 70 up to 90% [30–37] while 7 studies (46%) showed lower DC values [38–44]. Ten studies (66%) evaluated the DC by chemical analyses such as Fourier transform infrared spectroscopy (FTIR) [32, 33, 35, 37–39, 41–44] while 3 studies evaluated the DC using Raman spectroscopy [30, 31, 34], and another one performed near-infrared spectroscopy (NIRS) analyses [36]. Other 3 studies (20%) correlated flexural strength or hardness values with the DC [38, 39, 44]. Four studies (26%) evaluated the effects of resin-matrix composites thickness on the DC values. The commercially available resin-matrix cements assessed were: Panavia™ (Kuraray Noritake Dental Inc. Okayama, Japan); Mutli link™ (Ivoclar Vivadent, Schaan, Leichtenstein); Rely X™ (3M ESPE, Saint Paul, Minesotta, USA); Choice 2™ (Bisco, Inc. Schaumburg, IL, USA); Nexus 3™ (Kerr,Orange,USA), G-CEM LinkAce™ (GC Corp, Japan), and Enamel HFO™ (Micerium, Avegno, Italy) (Table 1). Most of resin-matrix composites revealed an organic matrix composed of Bis-GMA [30, 32, 33, 37, 44], UDMA [30–32, 35, 36, 39, 41–43], Bis-EMA [33], TEGDMA [30, 33–36, 39, 41–43], and camphorquinone (CQ)/tertiary amine photoinitiator system [35, 36, 40, 42]. The following micro- or nano-scale inorganic fillers were found in resin-matrix cements: amorphous silica, strontium glass, zirconium silicate, barium borosilicate glass, fluoro-aluminosilicate glass, sodium persulfate cupric acetate, and ytterbium fluoride (Table 1). The data recorded for resin-cement matrix, type of restoration, light curing parameters, degree of conversion and properties are given in Table 1.

**Table 1:** Relevant data gathered from the retrieved studies

Author (Year)	Study design/Purpose	Resin-matrix cements (chemical composition)	Restoration type	Light-curing procedure/ Analyses	Degree of Conversion (%)	Main outcomes
Velo et al. (2019) [38]	In vitro study. Assessment of the physical properties of hybrid nanofibers embedded with niobium pentoxide (Nb_2_O_5_) were synthesized and incorporated in self-adhesive resin cement	Four formulations: 1) U200 resin cement (control); 2) U200 and 1 wt% PDLLA fibers; 3) U200 and 1 wt% Nb_2_O_5_-filled PDLLA composite fibers and 4) U200 and 1 wt% Nb_2_O_5_/SiO_2_-filled PDLLA inorganic–organic hybrid fibers. (RelyX U200, 3 M ESPE)	Indirect restoration	LED (Valo,Ultradent) 1000 mW/cm^2^ for 20 s at 1 mm distance Analyses: DSC, 3-point bending strength, Knoop hardness measurement, FTIR-ATR	1) 67.6 2) 61.5 3) 63.2 4) 54.6	Increased hardness and flexural strength for hybrid nanofibers embedded with niobium pentoxide
Tomaselli et al. (2019) [30]	In vitro study. Evaluation of pre-heating, filler contents and ceramic thickness on film thickness, microshear bond strength, degree of conversion and color change on ceramic veneers.	Two experimental resin composites with a common matrix: Bis-GMA (29 wt%), UDMA (32. wt%), BisEMA (32.5 wt%), and TEGDMA (6wt%) 1) Resin composite (C) with 65 wt% of inorganic content: 13 wt% 0.05 μm fumed silica with 0.05 μm and 52 wt% 0.7 μm BaBSiO_2_ 2) Flowable composite(F) 50 wt% of inorganic content:10 wt% of 0.05 μm fumed silica and 40 wt% of 0.7 μm BaBSiO_2_. To both resins 0.25 wt% of camphorquinone	Disk shaped ceramics with thickness of 0.4, 0.8 and 1.4 mm	LED (Bluephase G2, Ivoclar Vivadent) 1200mW/cm^2^ for 20 s Analyses: micro shear bond strength, FT-Raman spectroscopy, Light spectrometry	1) 80.43 2) 77.92 CPH 77.29	Showed high color change. All composites demonstrated similar microshear bond strength
Lise et al. (2017) [31]	In vitro study. Assessment of light irradiance (LI) delivered by two light-curing units (LCU’s) and to measure the degree of conversion (DC) of three composite cements, when cured through different thicknesses of two novel CAD–CAM block materials.	1) Dual cure self-adhesive composite cement-G-CEM LinkAce, GC. Paste A: UDMA (10–20%) Y-methacryloxypropyltrimethoxysilane (> 2.5%). Paste B: UDMA (25–50%), methacryloxypropyltrimethoxysilane (> 2.5 to 10%), A, α-dimethylbenzylhydroperoxide 2) Flowable resin-based (RBC) G-ænial Universal Flo, GC. Filler (69 wt%): silicone dioxide (16 nm), strontium glass (200 nm). Matrix: UDMA, Bis-MEPP, TEGDMA Others: pigment, photoinitiator 3) Microhybrid RBC G-ænial Posterior, GC.Filler (81 wt%): fumed silica, fluoroaluminosilicate, strontium and lanthanoid glass. Matrix: UDMA, dimethacrylate monomers Others: pigments, catalysts	CAD–CAM blocks (Cerasmart, GC; Enamic, Vita Zahnfabrik) with 1-, 3- and 5-mm thickness	Two LED devices: -G-Light Prima (GC) and SmartLite Focus (Dentsply, York, PA, US Low irradiance (500 mW/cm^2^) High irradiance 1600 mw/cm^2^) Each specimen was light cured for 40 s Analyses: Light spectrometry, micro-Raman spectroscopy, STEM	43 to 80	Light-curable composite cements can be properly polymerized through CAD-CAM block materials with thickness of 1.5–2.7 mm, depending on the CAD-CAM block material type
Barbon et al. (2019) [39]	In vitro study. Investigating the influence of resin-based luting agents loaded with different inorganic filler content, with or without an adhesive.	Experimental resin-matrix luting agents; Matrix: UDMA and TEGDMA (1:1) Photoinitiator: camphorquinone (0.4 wt%) and ethyl 4-dimethylamino benzoate (0.8wt%) Inorganic phase: Barium borosilicate glass particles (2-mm average size) coated with 1wt% silane coupling agent were used as fillers A) low filler content (55 wt%) B) intermediate filler content (65 wt%) C) high filler content (75 wt%) Used as control with 66 wt% in filler content (RelyX Veneer, 3 M, USA)	Feldspathic ceramic blocks (VITABLOCSTM Mark II A1C, Vita Zahnfabrik GmbH, Germany)	LED (Radii; SDI) for 30 s at 1200 mW/cm^2^ Analyses: biaxial flexural strength, SEM, FTIR, micro tensile bond strength.	RelyX Veneer (control)—41.3 Low filler content (55wt%) – 52.7 Intermediate filler content (65wt%)- 52.6 High filler content (75wt%) 51.7	Experimental luting agent loaded with high inorganic filler content strengthened the bonded feldspathic ceramic and yielded significantly higher viscosity and film thickness.
De Kuijper et al. (2021) [32]	In vitro study. Investigating the influence of the ceramic translucency, restoration type and polymerization time on the relative degree of conversion of a dual-curing resin cement and a conventional microhybrid resin composite using a high-power light-curing device.	A) Dual-curing resin cement (DCC); Base: ytterbium trifluoride, UDMA, acetyl-2-thioureum Catalyst: ytterbium trifluoride, UDMA, acetyl-2-thioureum, α,α-dimethylbenzylhydroperoxide (Variolink Esthetic DC Warm; Ivoclar Vivadent; Schaan, Liechtenstein) B) Microhybrid composite (MHC); DUDMA, Bis-GMA; 1,4 – Butandioldimethacrylate (Enamel HFO UD3; Micerium; Avegno, Italy)	Lithium disilicate reinforced glass ceramics, CAD/CAM blocks (IPS e.max CAD A2 HT/ LT; Ivoclar Vivadent; Schaan, Liechtenstein)	LED (Bluephase 20i; wave length range: 385–515 nm; Ivoclar Vivadent; Schaan, Liechtenstein) 1200mW/cm^2^ Analyses: FTIR-ATR	0.1 to 99	DC for DCC cement was not significantly affected by the ceramic translucency or restoration type. DC for MHC cement was significantly lower for LT/EC than HT/EC restorations after 3 × 60 s polymerization. However, no differences were found for the high translucent restorations and low translucent onlays
Mendonça et al. (2019) [33]	In vitro study. Evaluation of the effect of the transmittance of ceramics with different composition, opacities and shades on the degree of conversion of two dual-cured resin cements	A) Bis-GMA, Bis-EMA, TEGDMA (34 wt%), Barium 1.5 alumo-silicate glass, silica fillers (66 wt% fillers) (Allcem, FGM, Brazil) Bis-GMA, UDMA and TEGDMA (28wt%), Barium glass, ytterbium trifluoride, Ba-Al-fluorosilicate glass, and silica fillers (72 wt% fillers), (Variolink II, Ivoclar Vivadent, Liechtenstein) B) Methacrylate monomers containing phosphoric acid groups, methacrylate monomers (28 wt%), silanated fillers, alkaline fillers 0(72 wt% fillers) (RelyX U200, 3 M, USA)	Lithium disilicate-reinforced glass ceramic (IPS e.max® Press, Ivoclar Vivadent AG; Schaan, Liechtenstein). Zirconia (Z) (IPS e.max® ZirCAD, Ivoclar Vivadent AG; Schaan, Liechtenstein)	LED (DB 685, Dabi Atlante; Ribeirão Preto, São Paulo, Brazil); 1400 mW/cm^2^ Analyses: Light spectrometry, FTIR-ATR	50 to 70%	The transmittance and DC values of the cements were influenced by the chemical composition and shades of the ceramics. The higher the transmittance of ceramics, the higher the DC values for both cements
Hardy et al. (2018) [34]	In vitro study. Evaluation of the limitations of using light-curable resin-based luting composites (RBLCs) to bond indirect ceramic/resin-composite restorations by measuring light transmittance through indirect restorative materials and the resulting degree of conversion (DC) of the luting-composites placed underneath.	Four experimental formulations Matrix: TEGDMA and BisGMA at 50/50 and 30/70 wt% ratios Phtoinitiators: camphorquinone/amine (0.2/0.8 wt% or Lucirin-TPO (0.42 wt% – Lu-TPO) Inorganic phase: microfillers (55 wt%) and nanofillers (10 wt%) Control Matrix: BisGMA 10–20 wt % and TEGDMA 10–20 wt%. Photoinitiators: Titanium Dioxide < 1 wt%) Ethyl 4-Dimethyl Aminobenzoate (EDMAB) (< 1 wt%) Benzotriazol(< 1 wt%) Diphenyliodonium Hexafluorophosphate (< 1 wt%) Inorganic phase: Silane treated ceramic (55–65 wt%), Silane treated silica (1–10 wt%) and Reacted polycaprolactone polymer (1–10wt%) RelyX Veneer (3 M-ESPE, St Paul, MN, USA)	Zirconia CAD-CAM blocks and LAVA Ultimate	AURA, Lumencor, USA; curing tip diameter = 6 mm; 1000 mW/cm^2^ or dual-peak. BluephaseG2 (BPG2, Ivoclar-Vivadent, Schaan, Liechtenstein; curing tip diameter = 10 mm; “High power”) Analyses: Light spectrometry, Raman spectroscopy	0.10 to 74.73	Under specific conditions, optimal polymerization of s resin cements could be achieved through indirect restorative materials (≤ 4 mm) and an irradiation time of 40 s
Castellanos et al. (2019) [35]	In vitro study. Evaluation of the light transmittance of ceramic veneers of different thickness and verify their influence on the degree of conversion, color stability, and dentin bond strength of light-curing resin cements containing different photoinitiator systems.	Four experimental light-curing luting resin cements with different photoinitiator concentrations: Matrix: BisGMA (20 wt%), UDMA (10 wt%), TEGDMA (10 wt%) Inorganic phase 60 wt% of: silica (10 wt%) with 0.05 μm and BaBSiO_2_ glass (50 wt%) with 0.7 μm Photoinitiator: A) camphorquinone (CQ) 0.2wt%- CQ-amine ratio 1:1 B) TPO-1:0 C) Ivocerin- 1:0 D) Ivocerin + TPO- 0.5 + 0.5	Disk-shaped lithium disilicate-reinforced glass ceramic. IPS e.max Press (Ivoclar Vivadent, shade LT/A2, Liechtenstein)	LED (Bluephase G2, Ivoclar Vivadent, Liechtenstein) Analyses: Light spectrometry, FTIR, micro-shear bond strength	52.25 to 72.78	Amine-free cements containing Ivocerin and TPO seem to be a better alternative to CQ-amine cements, while not reducing either DC or dentin μSBS of amine-free cements. However, CQ amine and amine free cements still seem to change color over time.
Franken et al. (2019) [40]	In vitro study. Development of experimental light-cured (L) and dual-cured (D) resin cements containing N-(2-hydroxyethyl)acrylamide and evaluation of the physicochemical and optical properties	Three experimental light-cured resin cements and six dual cured were formulated: Matrix: BisGMA (70%) HEMA (30%), and, BisGMA (70%) and HEAA(30%) Photoinitiator: CQ + EDAB on light cured cements, and CQ + benzoyl peroxide and DHEPT and the inhibitor BHT Inorganic phase:Ytterbium trifluoride (40 wt%)	Premilled lithium disilicate-reinforced glass ceramic blocks (IPS e.max CAD; Ivoclar Vivadent, Schaan, Liechtenstein)	LED (Radii Cal., SDI Ltd., Melbourne, Australia); 1200 mW/cm^2^ Analyses: DSC, Knoop hardness tests, X-ray images, light spectrometry, micro-shear bond strength, tensile strength	31.2 to 49.4	The addition of N-(2-hydroxyethyl)acrylamide negatively affected the properties of the dual-cured resin cements. LHEAA1 did not differ in physicochemical and optical properties from the control, with higher hydrolytic stability.
De Almeida et al. (2018) [41]	In vitro study. Evaluation of the shear bond strength (SBS) of self-adhesive resin cements (SARCs) to dentin and their physicochemical properties.	A) Matrix: UDMA, EBPADMA Urethane Resin, di-and tri- functional diluentes, PENTA. Photoinitiator:n/a Inorganic phase: 69% wt% of fillera (SmartCem2) B) Matrix: Bis (Hydroxyethyl methacrylate) phosphate, tetraethylene glycol dimethacrylate Inorganic phase: dental glass (Biscem) C) Matrix: UDMA, camphorquinone, acidic monomer Inorganic phase: Fluoro-aluminosilicate glass, (SeT PP) D) Matrix: methacrylated phosphoric acid esters, TEGDMA Inorganic phase: Glass powder, silane treated silica, sodium persulfate cupric acetate (Relyx U100) E) Dimethacrylate, acidic monomer, glass particle, silica nanoparticle; B: Dimethacrylate, initiators, glass particle, silica nanoparticle (YCEM AS-A)	Indirect restoration	LED (Radii; SDI, Bayswater, Australia) with 1000 mW/cm^2^ for 30 s Analyses: shear bond strength, pH, FTIR-ATR, 3-point bending strength test	12.42 to 38.76	A significant decrease in SBS was found for all groups after 12 months. SBS was not correlated with physicochemical properties, and appeared to be material-dependent. The polymerization profile suggested that an increased time of light activation, longer than that recommended by manufacturers, would be necessary to optimize DC of SARCs
Albuquerque et al. (2019) [36]	In vitro study. Evaluating the DC, physicochemical properties, and microshear bond strength of experimental self-adhesive resin cements to dentin and yttria-stabilized tetragonal zirconia polycrystals ceramic	Dural-curing cements were formulated SARCS Base: UDMA:HEMA 3:1 (36%), CQ (0.8%), EDAB (1.6%), DHPT (1%), BAS (1%) Catalyst: UDMA (10%), BisGMA (5%), TEGDMA (5%), HEMA (5%), BAPO (2%) Self adhesive characteristics Self adhesive characteristics: 2MP, GDMAP Inorganic phase: Base- glass fillers with 59 wt% and catalyst with 48 wt% in glass fillers Control cement Base: UDMA:HEMA 3:1, CQ (0.8%),EDAB (1.6%), DHPT (1%) (36%).Catalyst: UDMA (20%),BisGMA (10%),TEGDMA (10%) and HEMA (10%), BPO Self adhesive characteristics: 2MP, GDMAP Inorganic phase: Base- 60 wt% of glass fillers and catalyst 49 wt% of glass fillers	Pre-sintered Y-TZP blocks (In-ceram,YZ,VITA Zahnfabrik, Germany)	LED (Radii; SDI, Bayswater, Australia) with 1200 mW/cm^2^ for 20 s Analyses: FTIR, pH, micro-shear bond strength test	50.7 to 92.0	The results of DC, pH, Wsp and Wsi were material dependent. Only the film thickness was statistically similar in all groups. The cement formulated with GDMAP maintained the bond strength to dentin after aging.
Scotti et al. (2016) [42]	In vitro study. Evaluation of the degree of conversion (DC) and microhardness (MH) of a dual curing cement under lithium disilicate disks of different thickness.	1) Matrix: BisGMA, UDMA, EBPADMA, and TEGDMA, GPDM Photoinitiator: CQ Inorganic phase: 67.5 wt% of fillers, such as: bariumaluminosilicate glass filler, nanosized ytterbium fluoride filler, colloidal silica. (NX3, Kerr, USA) 2) Matrix: BisGMA (10%) Inorganic phase: strontium glass (75%), amorphous silica (25%) strontium glass, concentration range < 75%, amorphous silica, concentration range < 25%, bisGMA, concentration range < 10% (Choice 2, Bisco, USA)	Lithium disilicate-reinforced glass ceramics veneres (IPS e.max CAD, Ivoclar Vivadent, Liechtenstein)	LED (VALO, Ultradent) for 60 s at 1400 mW/cm^2^ Analyses: FTIR, Vickers hardness measurement	42.7 to 60.9	The light-curing and the dual curing cements reached comparable DCs between 0.6 and 1.5 mm.However, the light-curied resin composites showed a higher DC and MH
Kim et al. (2013) [43]	In vitro study. Measurement of the degree of conversion (DC) of dual-cured resin cements light-irradiated through zirconia ceramic disks with different thickness using various light-curing methods.	A) Base paste: BisGMA,TEGDMA,UDMA,glass filler Catalyst Paste: Bis-GMA, TEGDMA, glass filler (Duo-Link, Bisco, USA) B) Paste A: dimethacylates, 10-MDP, camphorquinone, catalysts, initiators, silanated silica filler, silanated colloidal silica Paste B: dimethacrylates, catalysts, accelerators, silanated barium glass filler, sodium fluoride (Panavia F 2.0, Kuraray, Japan)	Zirconia ceramic disks	LED (Bluephase G2, Ivoclar Vivadent, Liechtenstein) at 1150mW/cm^2^ QTH (Elipar Trilight,3 M ESPE,Germany) at 750mW/cm^2^ Analyses: FTIR, light spectrometry	A) Duo link: 21.8–61.9 B) Panavia: 10.6–48	The polymerization of the dual-cured resin cements was significantly affected by the light-curing technique, even though the additional chemical polymerization mechanism worked effectively.
Ganjkar; Heshmat; Ahangari (2017) [37]	In vitro study. Evaluation of the effects of IPS Empress porcelain thickness on the degree of conversion of light-cure and dual-cure resin cements.	A) Matrix: BisGMA (10%) Inorganic phase: strontium glass (75%), amorphous silica (25%) strontium glass, concentration range < 75%, amorphous silica, concentration range < 25%, bisGMA, concentration range < 10% (Choice2, Bisco, USA) B) Matrix: Bis-GMA (Nexus3, Kerr, USA)	IPS Empress ceramic (Ivoclar Vivadent, Liechtenstein)	LED (LEDemetron II; Kerr, Orange, CA USA) with 600 mW/cm^2^ for 40 s Analyses: FTIR, light spectrometry	A) 68.6 to B)72.5	It seems that increasing the porcelain thickness by up to 1.5 mm has no adverse effect on degree of conversion of both dual cure and light cure resin cements evaluated in this study
Cho et al. (2015) [44]	In vitro study. Evaluation of the effects of ceramic veneer thicknesses on the polymerization of two different resin cements.	A) Bis-GMA and Dimethacrylate (Nexus3, Kerr, USA) B) Bis-GMA and Dimethacrylate (Nexus 3 DC, Kerr, USA)	Ceramic veneer e.max Ivoclar Vivadent	LED (Demi Plus LED; Kerr, USA) for 15 s Analyses: FTIR-ATP, light spectrometry, Vickers hardness measurement	14 to 34.8	The degree of conversion and hardness of the resin cement was unaffected on veneering thickness between 0.3 and 0.9 mm.

Organic compounds: Bisphenol A-glycidyl dimethacrylate (Bis-GMA), urethane dimethacrylate (UDMA), and triethylene glycol dimethacrylate (TEGDMA), Ethoxy- lated bisphenol A dimethacrylate (Bis-EMA), butylated hydroxytoluene (BHT), camphorquinone (CQ), dimethacrylate (DMA), Hydroxyethylmethacrylate (HEMA)

The main findings are described as follow.

- Selected studies revealed that some resin-matrix cements composed mainly of UDMA:HEMA 3:1 (36%) and TEGDMA (5%) reached the highest degree of conversion (DC) after 24 h [36]. The highest DC mean values acquired by FTIR analyses were recorded at 92 and 95% for Variolink™ and Enamel HFO™ [32, 36] followed by DC mean values ranging between 77 and 80% for two experimental flowable resin composites and a dual cure self-adhesive composite cement-G-CEM LinkAce™ [30, 31]. The lowest DC mean values were recorded at 10 and 38% by FTIR [41, 43, 44] and ranging from 30 up to 40% by NIRS [40];
- The auto-polymerizing pathway itself was not enough to ensure high hardness mean values of resin-matrix cements. Dual-cured resin-matrix cements showed higher hardness mean values (10.7) when compared with light-cured cements (1.25) [44]. Resin-matrix cements containing spherical-shape particles showed higher flexural strength (120–129 MPa), flexural modulus (12–15 GPa), and hardness (101–117 HV) mean values than those recorded for resin-matrix cements with irregular-shape particles [39]. The addition of hybrid nano-scale fibers filled with Nb_2_O_5_ and Nb_2_O_5_/SiO_2_ into the U200™ self-adhesive resin-matrix cement showed higher values of flexural strength at 66 MPa when compared with the control groups, 42.3 MPa and 40 MPa [38];
- DC mean values of resin-matrix cements light-cured through a ceramic veneer with 0.4 mm thickness was higher (83%) than those recorded for resin-matrix cements light-cured through a thicker ceramic layer of 1.5 mm (77.8%) [30]. Light attenuation and DC were significantly influenced by increasing thickness of the indirect restoration up to 5 mm and type of overlying material [31, 35]. Also, an adequate DC percentage was recorded at 61 and 54.6% for light-curing and dual-curing resin cements after polymerization underneath lithium disilicate-reinforced glass ceramics with thickness at 0.6 and 1.5 mm [42].

## Discussion

The present integrative review reported the main findings of relevant previous studies, taking into account the degree of conversion (DC) of several resin-matrix cements used in dentistry. A large variation of DC values was recorded for commercially available resin-matrix cements and therefore physical properties were also affected. The variation of DC values was dependent on the inorganic fillers, organic matrix content, restorative materials, and polymerization mode. Thus, the findings of the selected studies validate the hypothesis of the present review. A detailed discussion of the main factors that affects the polymerization of resin-matrix cements is provided as follows.

### Resin-matrix cements in dentistry

**Fig. 2:**
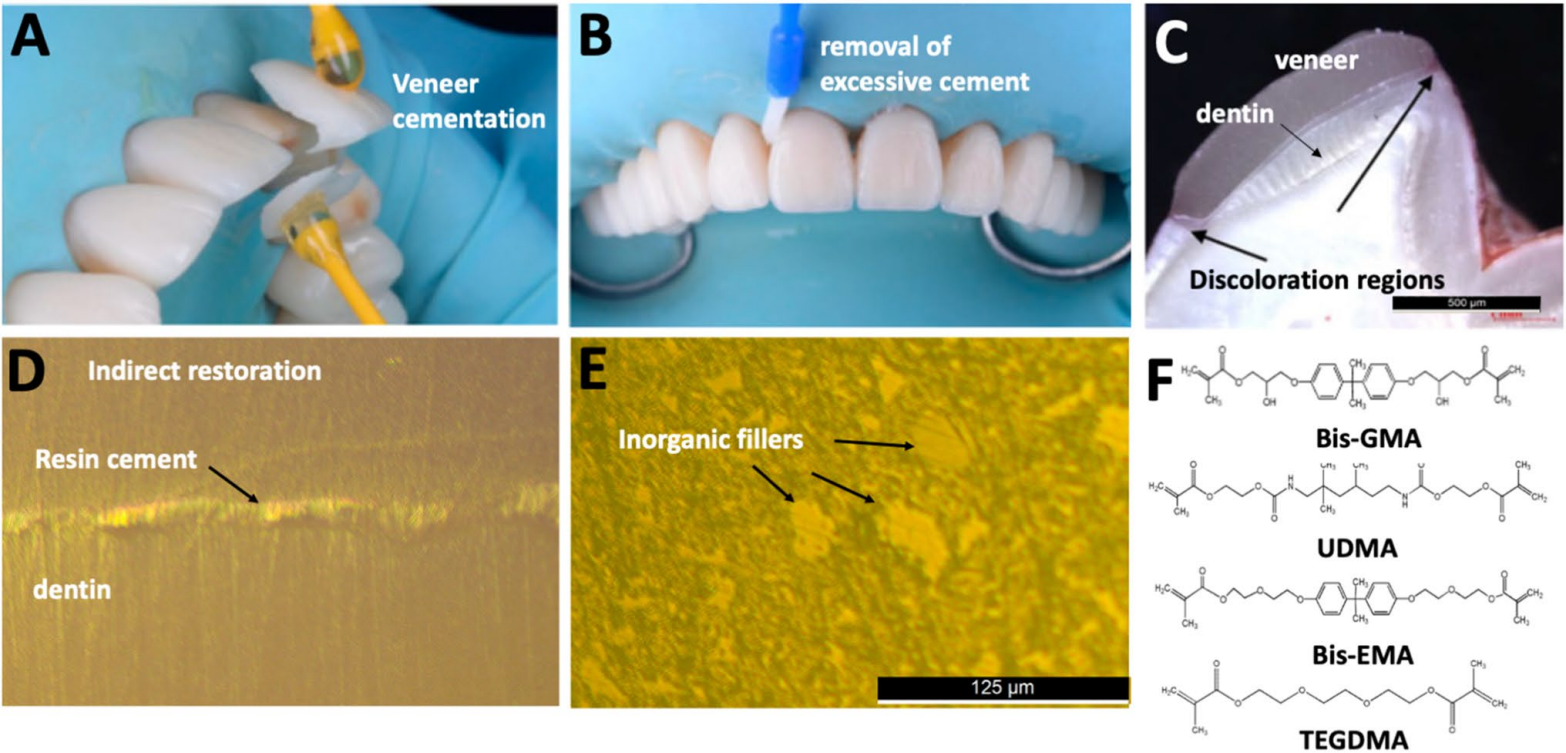
(**A**) Cementation and (**B**) removal of excessive layer of resin-matrix cements. (**C**) Clinical issues by discoloration at the restoration margins. Resin-matrix cement (**D**) interface and (**E**) microstructure. (**F**) Chemical composition of the organic matrix. Images adapted from [29, 45, 46]

Resin-matrix cements are widely used for luting of indirect restorations including onlays, inlays, crowns, and intraradicular posts (Fig. 2) [47, 48]. The resin-matrix composites possess the following properties which are important for cementing ceramic veneers: low solubility, translucency, flowability, and elasticity [23, 24, 49]. The chemical composition of resin-matrix cements involves the presence of Bis-GMA, TEGDMA, Bis-EMA and UDMA embedding silanized inorganic fillers such as colloidal silica, ytterbium, or barium glass as seen in Fig. 2E and F. The chemical composition of the resin-matrix cements also varies in the presence or not of photoinitiator compounds [27, 29, 33, 50]. For light-activated curing, photoinitiator compounds such as camphorquinone coupled to a tertiary amine in the organic matrix are stimulated by the light irradiance at a wavelength ranging from 420 up to 490 nm [51–54]. Other photoinitiators can be associated with the camphorquinone or used separately such as 1-type is trimethylbenzoyl-diphenylphosphine oxide (TPO), benzoyl peroxide (BPO), phenanthrenequinone (PQ), benzophenone (BP), and 1-phenyl-1,2 propanodione (PPD), type I photoinitiators. However, previous studies have revealed changes in degree of conversion, polymerization shrinkage, color stability, and mechanical properties of resin-matrix composites containing novel photoinitiators [20, 55]. The type I photoinitiators have promoted properties optical properties when compared to CQ, although differences in chemical composition, thickness, shade, and opacity of indirect restorative materials can cause polymerization issues in deeper restorative areas [56–58]. A recent article [59] investigated the relationship between the ceramic veneers thickness, photoinitiators, and polymerization of resin-matrix cements, and conclude the light irradiance was attenuated under different thickness, mostly in violet spectrum, and consequently affecting the mechanical properties of resin-matrix cements.

Currently, resin-matrix cements involve a dual polymerization pathway under light-induced and chemical activation that are complementary [22, 43, 57]. The formulation of dual-cured resin cements includes a catalyst paste (chemical initiator) and a base paste (light-cured resin cement and tertiary amine) [2, 8, 20]. Traditional dual-cured resin-matrix cements requires a previous application of an adhesive system coating the tooth substrate. Self-adhesive resin cements can contain functional monomers for chemical bonding such as 10-methacryloyloxydecyl dihydrogen phosphate (10- MDP), 4-methacryloxyethyl trimellitate anhydride (4-META), or phosphoric esters (Table 1). Self-adhesive resin-matrix cements have acidic monomers to partially demineralize the smear layer structure [28, 29, 41]. Dual-cured resin-matrix cements reveal advantages over chemically-cured resin cements considering a high degree of conversion (DC) of monomers, gradual shrinkage stresses, and feasible working time for chair-side procedures [23, 26, 29, 41, 43, 60]. However, light-cured resin-matrix cements are indicated beneath thin veneers since the light transmission is required for their polymerization pathway [23, 24, 26, 28, 31].

Thus, the organic matrix of resin-matrix cements is reinforced by inorganic fillers, with a weight content ranging from 36 to 87% [60–64]. Those inorganic particles revealed irregular- or rounded-shape morphological aspects and size at micro-scale (0.1 up to 10 µm) and nano-scale (20–60 nm) depending on the manufacturing [65–67]. A high filler content provides a high strength of the resin-matrix cement that enhances the mechanical properties of the interface [23, 24, 29]. However, the content and type of fillers content and optical properties also affect light transmittance under polymerization. A high content of inorganic fillers can negatively affect the light transmittance under light curing although it also depends on the size and chemical composition of the inorganic fillers [38, 41, 68]. The refractive indexes of the organic matrix and the inorganic fillers should be balanced to allow the light transmittance through the resin-matrix cements [63, 69].

A high content of nano- and micro-scale inorganic particles results in a low organic matrix volume under polymerization. The size and the content of inorganic fillers highly enhanced the strength and elastic modulus in resin-matrix composites [20, 24, 38, 39]. Thus, the particle size distribution into resin-matrix cements plays a key role on the performance of the materials. Variations in particle size influences tensile bond strength, shear bond strength, and marginal fitting to dentin [20, 24, 29, 38, 39, 51]. The organic content of resin-matrix cements is susceptible to the absorption of water and fluids that also depends on the DC of monomers [24, 29]. The DC of monomers is influenced by the content, type and size of the inorganic fillers [24, 29, 51, 53]. Fillers’s content directly affects monomers conversion, due to the attenuation of light that is enhanced at higher filler content concerning light scattering [17, 24]. However, light transmission is affected by both fillers size and fillers amount. Higher filler amount tends to reduce light transmission due to the increased light refraction at the interfaces between filler particles and resin matrix although the type of inorganic particles also influences the light behavior [17, 24, 29]. Thus, filler particles with high refractive indexes, such zirconia (e.g.) allow higher light transmission values, which is critical for light-curing materials such as dual-cured resin-matrix cements [24, 29, 32, 51].

### Degree of conversion of monomers in the resin-matrix cement

Adequate polymerization is fundamental to gathering physicochemical properties of the resin-matrix cements leading to an optimal stability of color and chemical composition [70]. Degree of conversion (DC) of monomers refers to the conversion of monomeric carbon–carbon double bonds into polymeric carbon–carbon single bonds [8, 71–76]. The DC provides a significant status of the mechanical performance of resin-matrix materials since an adequate polymerization results in enhanced physical properties [8, 9, 22, 24, 77–80].

**Fig. 3:**
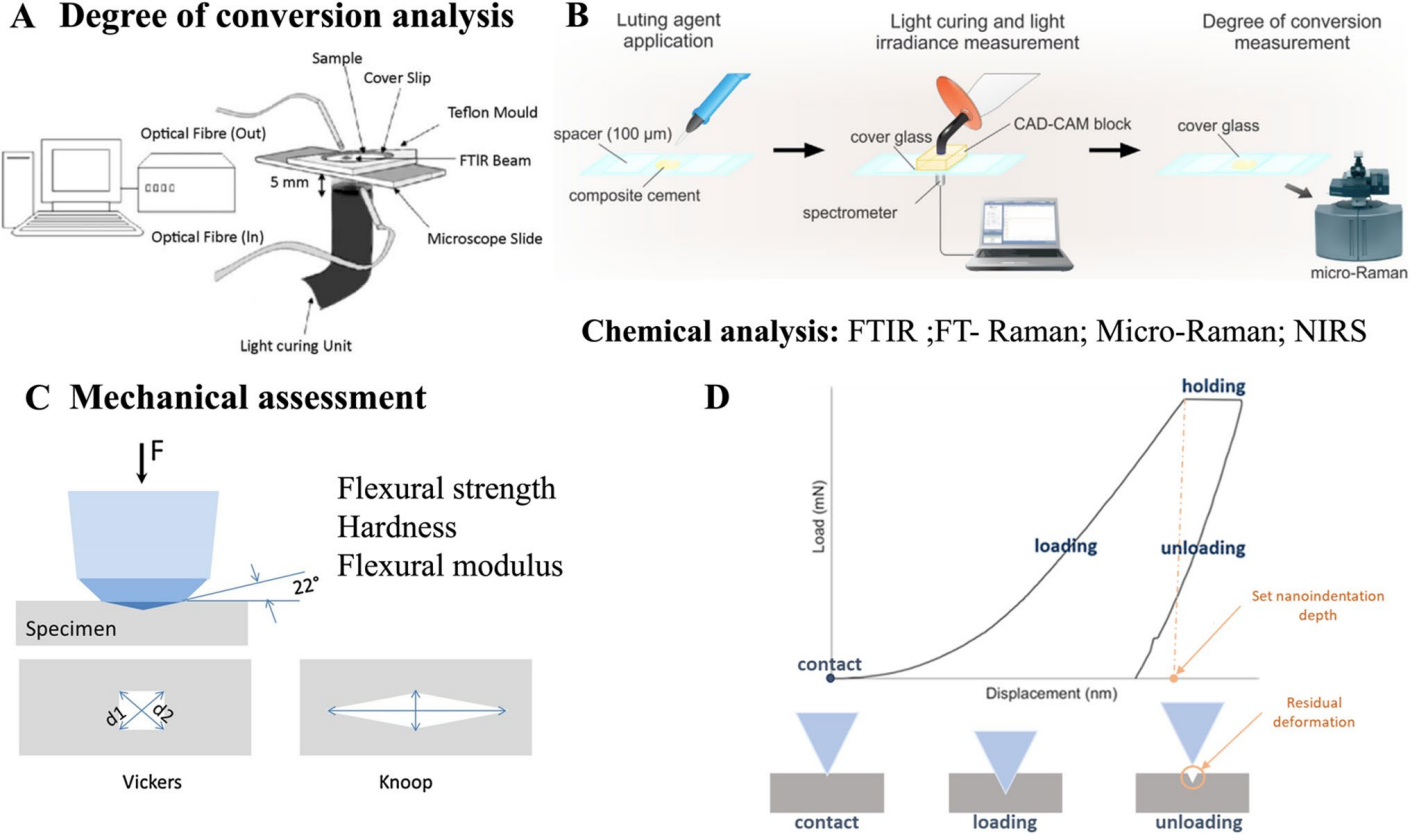
Set up for (**A**, **B**) light transmittance through materials. (**C**, **D**) Mechanical assessment of the materials by nanoindentation tests. Adapted from [31, 34, 54, 61, 81]

Several methods have been used to assess the DC, such as FTIR spectroscopy as shown in the selected studies given in Table 1 and illustrated in Fig. 3. FTIR spectroscopy has been used widely as an appropriate and reliable method. FTIR spectrometer detects C = C stretching vibrations and therefore such method has been used directly before and after polymerization of resin-matrix materials [33, 35, 41, 82–84]. There are multiple techniques that quantify DC of monomers that include mid-and-near infrared spectroscopy and Raman spectroscopy, Table 1 [30, 31, 34, 85]. FTIR is the most common analytical method to determine DC, the mid-IR spectrum (∼ 4000–400 cm^−1^ that corresponds to 2500–25,000 nm) contains several fundamental absorbance bands associated with the carbon–carbon double bond in methacrylate monomers [85–87]. The near-infrared spectroscopy is also used for DC assessment, at a spectrum ranging 4000 up to 12,500 cm^−1^ (2500–800 nm), providing a direct measurement of the methacrylate groups and other reactive groups. Raman spectroscopy, like FTIR allows the measurement of reactive groups concentration during and following polymerization, although the measurement involves light scattering rather than light absorption. Also, Raman spectroscopy is usually applied to measure DC polymerization quality in adhesive layers [85].

High energy density (20 J/cm^2^) derived from both increased light curing time and low irradiance from the LCU results in a faster release of free radicals and, consequently, a higher DC of the resin-matrix cements [22, 88]. Previous findings reported that an increase in light exposure time associated with low irradiance improved DC and the polymerization depth due to the delay in the formation of rigid grid bonds between polymer chains [42]. DC percentage in the organic matrix depends on the light curing unit (LCU) irradiance, light wavelength, light exposure time, light transmission, and light absorption by the photoinitiator [8, 24, 35, 51–54]. Several studies have reported that DC values should be higher than 55% on occlusal restorations [33, 35, 41, 42, 54, 68, 89]. DC is also dependent on the filler volume, fraction, particle size, shape and the refractive index [24]. Inorganic fillers that have refractive index close to monomers of the organic matrix, allow better light transmittance, which in turn influences the DC of resin cements [24, 29, 88]. It is also worth to mention that the type of monomers within the composition of resin matrix cement, beyond the refractive index compatibility, must demonstrate the right viscosity that favors the monomers mobility and also the final degree of conversion [8, 50]. Bis-GMA is known as monomer with increased viscosity and then diluent monomers (e.g., TEGDMA) are added to the Bis-GMA organic matrix formulations to decrease the viscosity and improve monomers cross-link. However Bis-GMA is still used in resin cements formulations due to adequate overall mechanical properties. UDMA is a monomer with more flexibility and crosslinking capacity and it can be incorporated with Bis-GMA in organic matrix formulations [14, 90]. The DC of resin cements with Bis-GMA in their composition is dependent on the amount of TEGDMA, that improves the degree of conversion because of its mobility and reactivity [87]. Polymerization of resin-based materials depends on chemical composition and refractive indexes of both inorganic and organic components [23, 29, 91].

Regarding the translucency, it has been reported that the higher the opacity and saturation of the resin-matrix cement decreases the light transmission leading to poor physicochemical properties [31, 43, 53, 92]. Thus, use of higher opacity ceramics is required in clinical cases to mimic the optical properties of the surrounding teeth although the light curing time should be extended to provide a required amount of energy for polymerization [3, 13, 22–24, 70]. The evaluation of the DC by keeping light curing unit at a 6-mm distance from the top surface of the specimen is clinically relevant because it simulates what happen to DC in difficult-to-access posterior restorations [88].

The light irradiance under light curing arriving at the luting agent is also affected by the indirect restoration [31, 34, 43, 54, 92]. The thickness and translucency of the ceramic restoration affect the light transmittance towards the resin-matrix cement [13, 34, 35, 37, 40, 42, 44, 92]. The amount of light irradiating through the zirconia or glass–ceramic prosthetic structures depends on the translucency, microstructure, chemical composition, irradiation energy, thickness, porosity, and manufacturing technique [55, 93, 94]. Glass–ceramic materials such as lithium disilicate-reinforced glass ceramics are translucent although a thick structure decreases the light transmittance towards the resin-matrix cement [3, 23, 24]. Considering the zirconia structures, fully stabilized zirconia is more translucent than partially stabilized zirconia. Indeed, the translucency of the prosthetic structures are mainly affected by their thickness and by the resin-matrix cement itself since a high translucency is achieved within thin zirconia or glass–ceramic veneers associated with resin-matrix cement [3, 23, 31]. It has been reported viable methods for cementing higher translucent glass ceramics up to 2-mm thick using a combination of high light irradiance and exposure time on the light-curing procedure [24, 29, 70].

In fact, the DC percentage of monomers varies regarding the type of resin-matrix cements and the mode of polymerization. There are many variables involved in the evaluation of DC for resin-matrix materials related to the prosthetic materials and resin-matrix cement itself. The limitations of this review study are resultant from the low number of studies comparing similar parameters such the chemical composition and microstructure of the resin-matrix cement and mode of polymerization. The light-curing unit (LCU) does not work at the same light irradiance comparing different in vitro studies and therefore the LCU should be cautiously monitored prior to the polymerization procedure. Clinicians should also be aware on the development of novel resin-matrix cement taking into account the type of photoinitiators and inorganic fillers. Then, the polymerization parameters (light irradiance, wavelength, and exposure time) can be optimized to achieve a high DC percentage of the organic matrix. Further studies should be carried out concerning the type and content of the organic matrix and inorganic fillers.

## Conclusions

Within the limitations of the selected studies, the main conclusions can be drawn as follow:

- Most of resin-matrix composites revealed an organic matrix composed of Bis-GMA, UDMA, Bis-EMA, and TEGDMA. The main photoinitiator system comprised camphorquinone (CQ) and tertiary amine while the chemical composition, size, and content of inorganic fillers varied comparing the resin-matrix cements. Thus, the refractive indexes of the organic matrix and the inorganic fillers should be balanced to allow a high light transmittance through the resin-matrix cements and providing the required energy for the degree of conversion of monomers.
- Most studies have reported a high degree of conversion (DC) of the organic matrix ranging from 70 up to 90%. Dual-curing resin-matrix cements showed the highest percentage of DC although a longer light exposure time is required for achieving an enhanced degree of conversion of monomers of the resin-matrix cement under prosthetic structures. A high degree of conversion of monomers resulted in enhanced physical properties of the resin-matrix cements. Dual-cured resin-matrix cements showed higher hardness mean values when compared with light-cured cements, showing that polymerization mode influences their mechanical properties.
- The thickness and microstructure of zirconia or glass–ceramic prosthetic structures play a key role on the light transmission towards the resin-matrix cement, and therefore directly influence DC percentage. Similar DC values for the same resin-matrix cement were recorded when the prosthetic structure showed a thickness below 1.5 mm. On thick prosthetic structures, translucent materials are required to allow the light transmission achieving the resin-matrix cement and guarantee high DC values. Thus, the equipment and procedures of light-curing must be carefully monitored by the clinician concerning the prosthetic structures and resin-matrix cements for optimal clinical outcomes.

## Data Availability

No datasets were generated or analysed during the current study.
